# Differential phosphofructokinase-1 isoenzyme patterns associated with glycolytic efficiency in human breast cancer and paracancer tissues

**DOI:** 10.3892/ol.2013.1599

**Published:** 2013-10-01

**Authors:** GUANNAN WANG, ZHILIANG XU, CHANGHUA WANG, FENG YAO, JUANJUAN LI, CHUANG CHEN, SHENGRONG SUN

**Affiliations:** 1 Department of Breast and Thyroid Surgery, Renmin Hospital, Wuhan University, Wuhan, Hubei, P.R. China; 2 Department of Pathology and Physiology, The Medical College, Wuhan University, Wuhan, Hubei, P.R. China

**Keywords:** glycolysis, phosphofructokinase-1, breast cancer

## Abstract

Cancers are characterized by an increasing glycolytic activity, which is called the Warburg effect. Although this phenomenon is well known, the mechanism of the enhanced rate of glycolysis in cancer has not yet been clearly recognized. The present study investigated the glycolytic rate, regulatory enzymatic activities and the expression of phosphofructokinase-1 (PFK-1) in human breast cancer and paracancer tissues. Human breast cancer tissues have an increased degree of glycolytic efficiency and regulatory enzymatic activities, which have been shown in previous studies. However, the present study identified a number of novel observations. The total PFK-1 levels were higher in human breast cancer tissues than in paracancer tissues, and further investigations revealed differential PFK-1 isoenzyme expression patterns between human breast cancer and paracancer tissues. The human breast cancer and paracancer tissues mainly expressed PFK-P and PFK-L isoforms, respectively. Linear-regression analysis showed that, depending on the pathological stage of breast cancer, the expression of PFK-P was significantly positively correlated with the activity of PFK-1. Thus, during the development of human breast cancer, the enhancement of glycolytic activity depends primarily on the conversion of the PFK-1, from PFK-L to PFK-P.

## Introduction

Malignant tumors, including breast cancers (1,2), usually exhibit a high rate of glycolytic activity compared with normal tissues in the presence of oxygen, known as the Warburg effect (3). This feature has been applied in a clinical setting, including positron emission tomography and computed tomography in oncology (4). Furthermore, the Warburg effect is considered to be a negative prognostic indicator, which may allow tumor cells to become invasive and develop a resistance to radiation and chemotherapies (5).

There are numerous mechanisms of the Warburg effect. Somatic mutations in mitochondrial DNA have been shown in a number of tumors. The outcome of such mutations is suboptimal or non-functional oxidative phosphorylation, meaning that cells must accelerate glycolysis (6). The second significant mechanism may involve hypoxia-inducible factor-1 (HIF-1), which is actively responsible for regulating the energy production in hypoxic cells. HIF-1 has been shown to induce the enzymes that are responsible for glycolysis (7,8) and to decrease mitochondrial respiration (9). Glucose transporter (GLUT) also plays a significant role in the Warburg effect. The transport of glucose is the first rate-limiting step for glucose metabolism and is mediated by facilitative GLUT proteins. An increase in glucose transport within malignant cells has been associated with an increased and deregulated expression of GLUT proteins, which provide adequate raw materials for glycolysis (10). Qualitative and quantitative changes in the regulatory glycolytic enzymes, hexokinase (HK), phosphofructokinase-1 (PFK-1) and pyruvate kinase (PK), are involved in the increase of the glycolytic flux (11). Among these glycolytic enzymes, PFK-1 has been more extensively studied than the others, which is likely to be due to its various regulatory mechanisms.

Human PFK-1 exists as three isoforms, PFK-M, PFK-L and PFK-P, which undergo random tetramerization to produce various homo- and heterotetrameric isoenzymes, which are distinguishable from one another by their kinetic properties (12). Thus, various tissues exhibit specific PFK-1 isoenzyme patterns that influence the glycolytic efficiency (13). Zancan *et al* reported the differences in PFK-1 isoenzyme patterns between the mRNA levels of non-tumorigenic and tumorigenic breast cells. PFK-L expression was identified to correlate with aggressiveness and glycolytic efficiency in these cell lines (14). The cellular distribution of PFK-1 activity has also been shown to have a key role in the regulation of metabolic activity. El-Bacha *et al* reported that the majority of PFK-1 activity in human breast cancer tissues is located in an actin-enriched fraction. Additionally, metastatic tumors, when compared with non-metastatic tumors, showed a significant increase in PFK-1 activity in this enriched fraction. The altered cellular distribution of PFK-1 activity in human breast cancer tissue may be associated with an increase in the glycolytic flux, which in turn is strongly associated with the process of carcinogenesis and tumor progression (11). Furthermore, Šmerc *et al* demonstrated that the post-translational modification of PFK-M in mammalian cancer cells consequently leads to the formation of active shorter PFK-M fragments with altered kinetic parameters, which may trigger the most significant change in the regulation of glycolytic flux in cancer cells and may also have an impact on the Warburg effect (15).

Despite a large number of studies to date, there have been no investigations with regard to the differences in PFK-1 isoenzyme patterns between human breast cancer and paracancer tissues. The present study compared the glycolytic efficiency and isoenzyme patterns of PFK-1 at the protein level in human breast cancer and paracancer tissues. It was found that the paracancer tissues shared the same genetic information and a similar microenvironment with the paired cancer tissues.

## Materials and methods

### Tissue procurement from patients

A total of 40 female patients, aged between 33–75 years, who were admitted to the Ren Min Hospital of Wuhan University (Wuhan, China) were recruited for this study (Table I). Patients with metabolic diseases such as diabetes mellitus and hyperthyroidism were excluded from this study. A total of 40 pairs of human breast cancer and paracancer tissues were obtained by dissection during surgery and were immediately placed into ice-cold normal saline. The samples were frozen in liquid nitrogen subsequent to being washed and trimmed. The characteristics of the tumor tissues were determined using contemporary histopathological examination of the fresh-frozen samples taken from three to five sites. The sections lying close to those that were tested histopathologically were dissected as tumor tissues. Samples of paracancer tissues that were not invaded by carcinoma were obtained from areas that were ~2 cm away from the tumors in order to avoid contamination by the disseminating cancer cells. The quality of these tissues was evaluated by conventional histological examination for a final histological diagnosis. Approval for this study was obtained from the Ethical Committee of Ren Min Hospital, Wuhan University and written informed consent was provided by the patients.

### Glycolytic enzyme activities assay

All specimens had a wet weight of ~50 mg following the addition of a 450-μl extraction buffer [1 M Tris (pH 7.5), 1% Triton X-100, 5 M NaCl, 50 mM EDTA, 100 mM PMSF, 0.5 M NaF and 100 mM Na_3_VO_4_]. Homogenization was performed in a Potter homogenizer (Beyotime Institute of Biology, Haimen, China) to prepare 10% homogenate (m/v). The homogenate was centrifuged for 10 min at 20,000 × g. The supernatant was used for the enzyme assays. All the procedures were carried out at 4°C.

As we were unable to perform the measurements instantaneously, the stabilities of the individual enzymes varied considerably in the homogenates, for this reason it was necessary to construct an enzyme assay program in which the most labile enzymes were measured first. Lactic acid (LA) content was measured first. The activity of the remaining enzymes was measured in the following order: PK, PFK-1, HK and then lactate dehydrogenase (LDH) (16).

The assay kits for the determination of the LA content (A019-2) and LDH (A020-1), HK (A077-1) and PK (A076-1) activity were purchased from Nanjing Jiancheng Bioengineering Institute (Nanjing, China). PFK-1 activity was assayed as described previously (17) in a medium containing 50 mM Tris-HCl (pH 7.4), 5 mM MgCl_2_, 5 mM (NH_4_)_2_SO_4_, 1 mM fructose 6-P, 1 mM ATP, 0.5 mM NADH, 2 mU/ml aldolase, 2 mU/ml triosephosphate isomerase, 2 mU/ml α-glycerophosphate dehydrogenase and 100 μl protein in a final volume of 1 ml. The reaction was initiated by the addition of the protein and NADH oxidation was recorded by measuring the decrease in absorbance at 340 nm using a spectrophotometer at 37°C.

The soluble protein content was measured using an assay kit purchased from Applygen Technologies Incorporation (Beijing, China).

One unit of activity was defined as the amount of enzyme that catalyzes the formation of 1 μmol of product per min in standardized conditions. Specific activities were expressed as units per gram of protein (U/gprot).

### Western blot analysis

Due to the low protein concentration of the human breast tissues in 10% homogenates, 20% (m/v) homogenates were prepared. The supernatant was obtained as described previously and boiled with 5X SDS-PAGE loading buffer at 100°C for 5 min. The prepared samples were subjected to 10% SDS-PAGE and transferred to polyvinylidene fluoride membranes (Millipore, Billerica, MA, USA). The membranes were then blocked in Tris-buffered saline, which contained 0.1% Tween 20 (TBST) and 5% confining liquid (rabbit serum or BSA) for 2 h at room temperature, followed by incubation overnight in the TBST containing the primary antibody at 4°C. Subsequent to being washed with TBST, the membranes were incubated with HRP-conjugated rabbit anti-goat antibody (Jackson ImmunoResearch, West Grove, PA, USA) or AP-conjugated mouse anti-rabbit antibody (Pierce, Rockford, IL, USA) for 1 h at room temperature. The antibodies that were used were anti-PFK-1 (catalog no SC-31711), anti-PFK-M (catalog no. SC-67028), anti-PFK-L (catalog no. SC-130226), anti-PFK-P (catalog no. SC-130227) and anti-β-actin (catalog no. SC-130300) (Santa Cruz Biotechnology Inc., Santa Cruz, CA, USA).

### Statistical analysis

The experimental data are presented as the mean ± SD. The statistical comparisons were performed by the SNK test, χ^2^ test or t-test, correspondingly, using SPSS 11.0 software (SPSS, Inc., Chicago, IL, USA). P<0.05 was considered to indicate a statistically significant difference.

## Results

### Common patient features

The patients were divided into three groups according to TNM classification (Table I). No significant differences were identified with regard to the mean age (SNK test; P=0.55, 0.86 and 0.43 in stage I vs. II, I vs. III and II vs. III, respectively) or hormonal activity (P=0.96; χ^2^, 0.090) of the patients in the various clinical stages.

### LA content and LDH activity are increased in human breast cancer tissues

**Figure 1:**
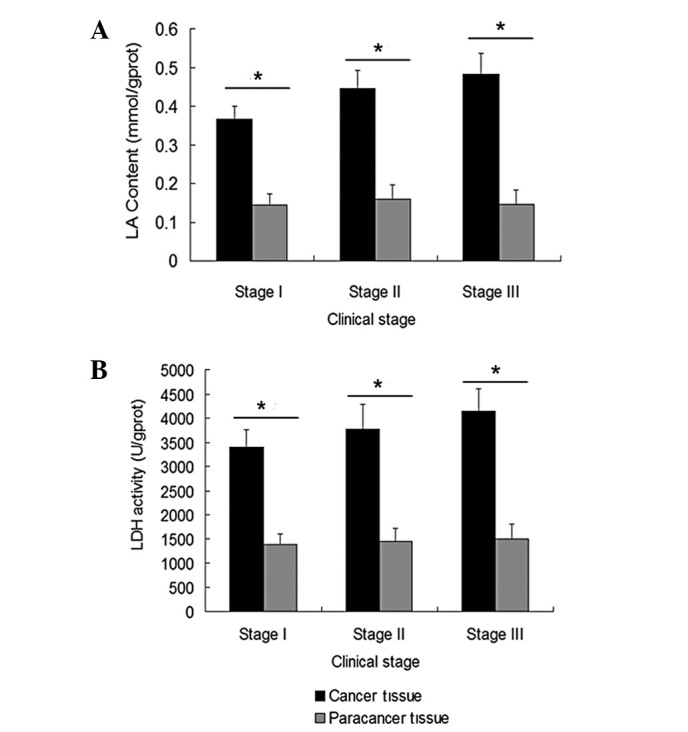
Lactic acid (LA) content and lactate dehydrogenase (LDH) activity in breast cancer and paracancer tissues. (A) LA content, expressed as mmol/gram of protein (mmol/gprot). (B) LDH activity, expressed as unit/gram of protein (U/gprot). ^*^P<0.05.

The LA content and LDH activity in human breast cancer and paracancer tissues were detected in order to evaluate the glycolytic efficiency. The LA content in the human breast cancer and paracancer tissues of each clinical stage (I, II and III) was 0.37±0.033 vs. 0.14±0.027 mmol/gprot (P=0.034), 0.45±0.047 vs. 0.16±0.036 mmol/gprot (P=0.027)and 0.48±0.052 vs. 0.15±0.036 mmol/gprot (P=0.017), respectively (Fig. 1A). The LDH activity in the human breast cancer and paracancer tissues of each clinical stage was 3399±352 vs. 1375±239 U/gprot (P=0.043), 3770±511 vs. 1449±267 U/gprot (P=0.035) and 4153±452 vs. 1499±310 U/gprot (P=0.029), respectively (Fig. 1B). Furthermore, the LA content and LDH activity in the breast cancer tissues increased with increasing clinical stage. Significant differences were identified in the LA content and LDH activity assays (P=0.042, 0.025 and 0.035 for LA and P=0.035, 0.020 and 0.031 for LDH in stage I vs. II, I vs. III and II vs. III, respectively).

### Activities of regulatory glycolytic enzymes are higher in human breast cancer tissues

**Figure 2:**
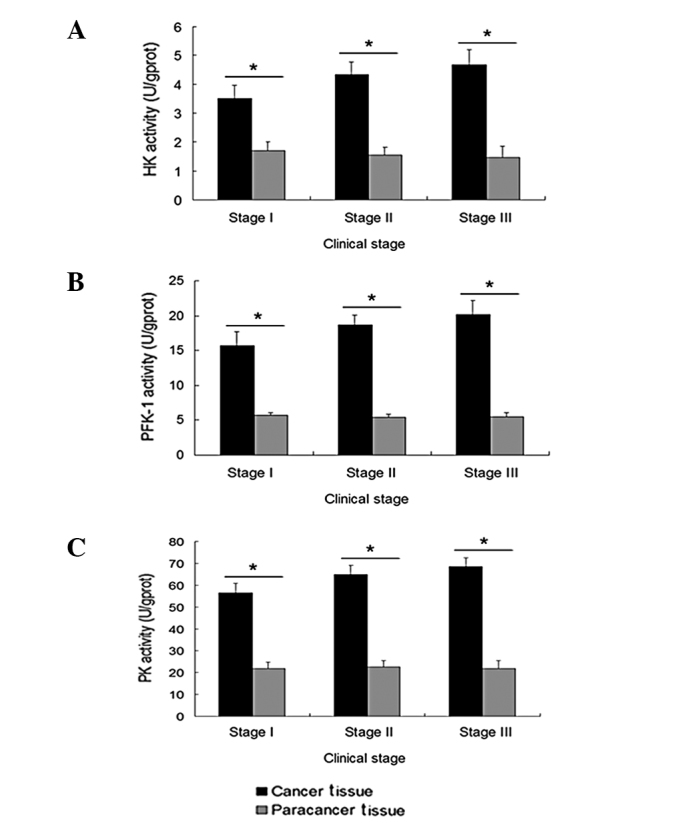
Activities of (A) hexokinase (HK), (B) phosphofructokinase-1 (PFK-1) and (C) pyruvate kinase (PK) in breast cancer and paracancer tissues, expressed as units per gram of protein (U/gprot). ^*^P<0.05.

HK, PFK-1 and PK regulate the rate of glycolysis. The activities of these enzymes were compared in order to identify which of these enzymes were responsible for the different rates of glycolysis between human breast cancer and paracancer tissues. The HK activities in the human breast cancer and paracancer tissues of each clinical stage (I, II and III) were 3.51±0.466 vs. 1.70±0.317 U/gprot (P=0.039), 4.34±0.422 vs. 1.55±0.260 U/gprot (P=0.022) and 4.68±0.518 vs. 1.47±0.389 U/gprot (P=0.011), respectively (Fig. 2A). The PFK-1 activities in the human breast cancer and paracancer tissues of each clinical stage were 15.7±1.92 vs. 5.71±0.366 U/gprot (P=0.024), 18.6±1.48 vs. 5.39±0.459 U/gprot (P=0.018) and 20.2±1.94 vs. 5.48±0.612 U/gprot (P=0.010), respectively (Fig. 2B). The PK activities in the human breast cancer and paracancer tissues of each clinical stage were 56.4±4.57 vs. 21.8±3.08 U/gprot (P=0.032), 64.9±4.07 vs. 22.5±3.03 U/gprot (P=0.021) and 68.7±3.92 vs. 21.9±3.58 U/gprot (P=0.012), respectively (Fig. 2C). The HK, PFK-1 and PK activities in the breast cancer tissues increased with increasing clinical stage. Significant differences were identified in the HK, PFK-1 and PK activity assays (P=0.041, 0.039 and 0.038 for HK, P=0.025, 0.018 and 0.027 for PFK-1 and P=0.033, 0.024 and 0.043, in stage I vs. II, I vs. III and II vs. III, respectively).

### Isoenzyme patterns of PFK-1 in human breast cancer and paracancer tissues

**Figure 3:**
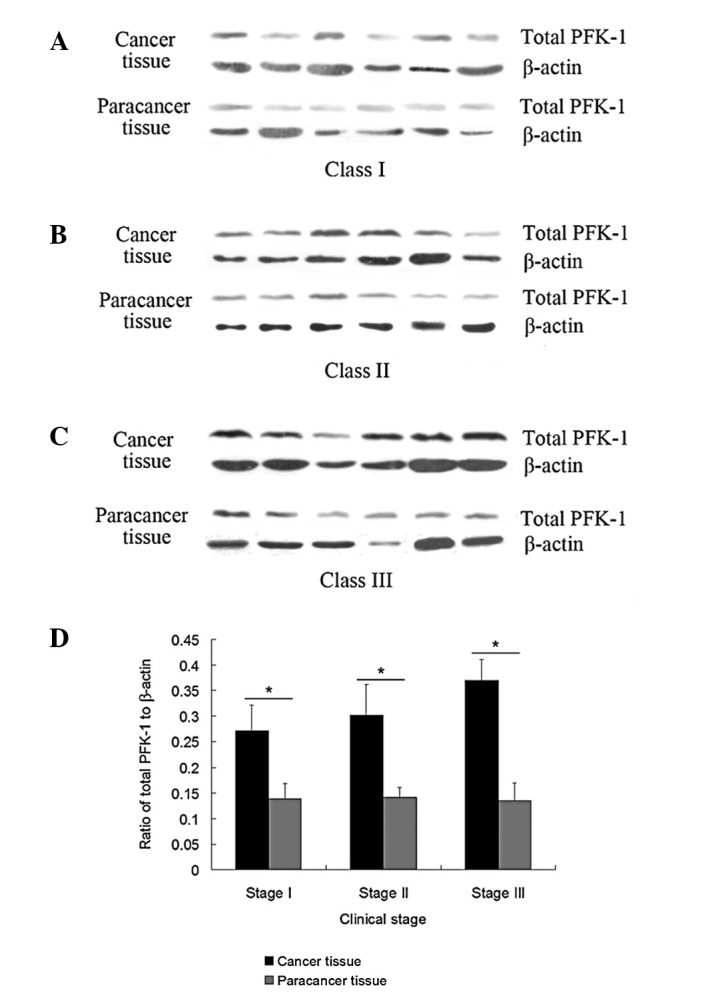
Expression of total phosphofructokinase-1 (PFK-1) in breast cancer and paracancer tissues. (A–C) Scanned images (representative) of total PFK-1 in human breast cancer and paracancer tissues of each clinical stage, by western blot analysis. (D) Relative quantity of total PFK-1 is expressed as the ratio of total PFK-1 to β-actin expression. The total PFK-1 expression was higher in human breast cancer tissues and in later clinical stages. ^*^P<0.05.

**Figure 4:**
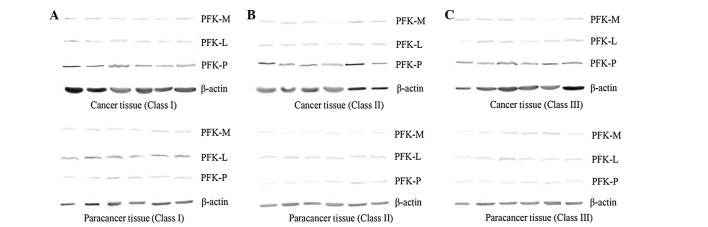
Expression of phosphofructokinase-1 (PFK-1) isoforms in breast cancer and paracancer tissues in each clinical group. (A–C) Scanned images (representative) of the PFK-1 isoforms in each clinical group, using western blot analysis.

**Figure 5:**
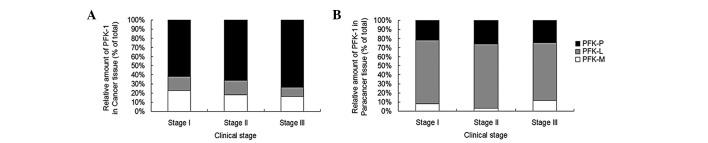
Phosphofructokinase-1 (PFK-1) isoenzyme patterns in (A) breast cancer and (B) paracancer tissues of each clinical group. PFK-1 isoenzyme patterns are expressed as ratios of each PFK-1 isoform to total PFK-1.

The enzymic activities in the malignant tissues were higher, particularly for PFK-1, as they were considered to be regulatory enzymes of glycolysis. To investigate the correlation between PFK-1 expression and glycolysis in breast tissues, the total PFK-1 content and PFK-1 isoenzyme patterns were evaluated using western blot analysis. The results are presented as scanned images (Fig. 3A–C) and as relative to β-actin (Fig. 3D). The ratios of total PFK-1 to β-actin expression were 0.272±0.051 vs. 0.139±0.032 (P=0.033), 0.303±0.064 vs. 0.142±0.022 (P=0.029) and 0.370±0.040 vs. 0.136±0.030 (P=0.018), respectively, between the human breast cancer and paracancer tissues of each clinical stage. The total PFK-1 content increased with increasing clinical stage, and significant differences were observed between stages I and II, I and III and II and III (P=0.032, 0.011 and 0.025, respectively). Furthermore, the isoenzyme patterns of PFK-1 were analyzed using western blot analysis, and significant differences were identified between the human breast cancer and paracancer tissues (Fig. 4). In the carcinomas, the percentages of the M, L and P isoforms of PFK-1 were 22, 16 and 62% in stage I, 18, 14 and 68% in stage II, and 17, 11 and 72% in stage III, respectively (Fig. 5A). By contrast, in the paracancer tissues, the percentages of the M, L and P isoforms of PFK-1 were 8, 70 and 22% in stage I, 3, 71 and 26% in stage II and 12, 64 and 24% in stage III, respectively (Fig. 5B). PFK-P and PFK-L accounted for the vast majority of the total PFK-1 content in the human breast cancer and paracancer tissues, respectively. The percentage of PFK-P increased with increasing clinical stage of the carcinomas.

### Correlation analysis between PFK-1 activity and isoenzyme patterns

**Figure 6:**
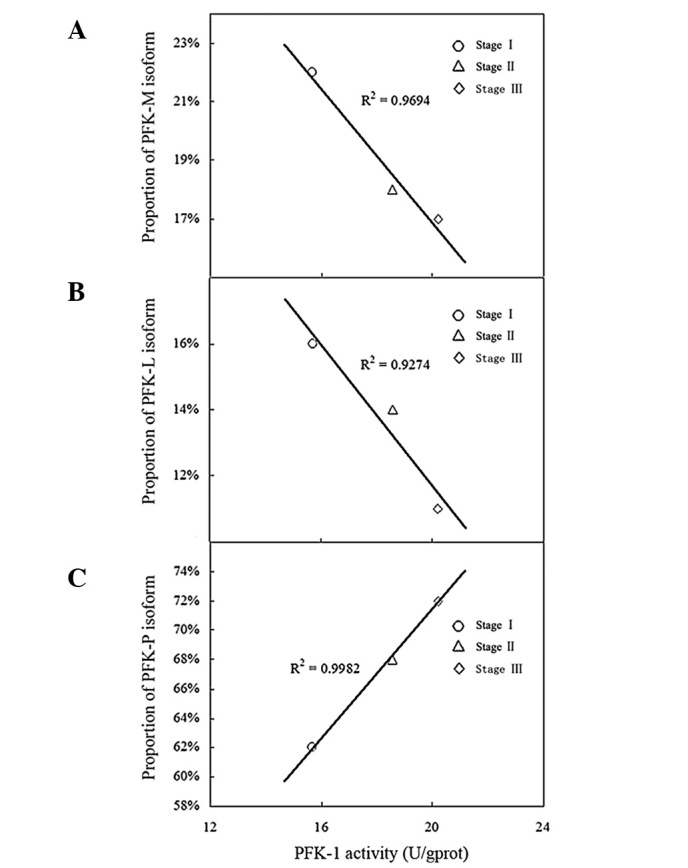
Correlation analysis between phosphofructokinase-1 (PFK-1) activity and isoenzyme patterns. Expression of (A) PFK-M and (B) PFK-L are negatively correlated with the activity of PFK-1. (C) Expression of PFK-P is positively correlated with the activity of PFK-1.

The correlation between PFK-1 activity and isoenzyme patterns was analyzed in the human breast cancer tissues of each clinical stage (Fig. 6). The statistics revealed that, with increasing pathological stages of breast cancer, the expression of PFK-P was significantly positively correlated with the activity of PFK-1 (R^2^=0.9982; P=0.032). The expression of PFK-M (R^2^=0.9694; P=0.107) and PFK-L (R^2^=0.9274; P=0.178) was negatively correlated with the activity of PFK-1, but without statistical significance.

## Discussion

The most well-known energy metabolism alteration in tumor cells is an increased glycolytic capacity in the presence of a high O_2_ concentration. A persistent metabolism of glucose to lactate, even in aerobic conditions, is an adaptation to intermittent hypoxia in pre-malignant lesions (18). Early studies on breast cancer have identified that the activities of all the glycolytic enzymes that were tested from carcinomas were significantly higher than those of non-malignant diseases (1). Glycolytic enzyme activities were significantly higher in breast cancer metastases compared with in primary tumors (19), and the transition of the breast cancer towards the normal surrounding breast tissue showed a decrease in glycolytic activity (20). However, the enhanced glycolytic rate requires further investigation in order to be completely understood.

Glycolytic enzyme activity has been reported to be age-related. In albino Swiss mouse liver tissue, the activity of PFK-1 was highest in 8- to 12-week-old mice, then gradually decreased with age. The PFK-1 activity was maintained at a stable level at 24 weeks old (21). Furthermore, a study has reported that the brain PFK-1 activity in the substantia nigra is lower in adult and aged mice (22). Indirect evidence has suggested that estrogen may affect the expression of the PFK-M subtypes. It has been identified that mouse PFK-M expression in the genioglossus increases in a state of chronic hypoxia, and estrogen is able to decrease PFK-M expression under hypoxic conditions (23). Although there is no direct evidence, the influence of age and estrogen levels may have an effect on breast cancer glycolysis. Therefore, the present study compared the average age and menstrual status between patients with various pathological stages of breast cancer. No significant differences were identified between the various stages of cancer and the age and menstrual status of the patients.

The glycolytic enzyme activity data presented in this study confirmed the results of earlier observations. It is well-known that LA is a product of the glycolysis process and LDH catalyzes the process of LA production (24). The present study evaluated the glycolytic rate by measuring the LA content and LDH activity. A significantly higher LA content and LDH activity was observed in the carcinomas compared with the paracancer tissues. Furthermore, the LA content and LDH activity in the breast cancer tissues increased with increasing clinical stage. The activities of the glycolytic regulatory enzymes, HK, PFK-1 and PK, in the human breast cancer tissues were all significantly higher than those in the paracancer tissues of each clinical stage. The enzymatic activities also progressed with the clinical stages.

The present study also detected the expression of total PFK-1 using western blot analysis. Due to the superior activity of PFK-1 in the cancer tissues, the results revealed that the total PFK-1 level in the human breast cancer tissues of superior clinical stages was higher. In order to further understand the mechanism of the increased glycolysis rate, the PFK-1 isoenzyme patterns were analyzed between the human breast cancer and paracancer tissues. Notably, a significant difference was identified. The human breast cancer and paracancer tissues mainly expressed PFK-P and PFK-L, respectively, which is concordant with the results of other studies. Sánchez-Martínez and Aragon observed that the presence of an ascites tumor of mammary origin predominantly contained PFK-P, whereas the PFK-L isoform was more abundant in the mammary gland. The proportions of the PFK-P, PFK-L and PFK-M isoforms in the ascites were 50, 32 and 18%, respectively; whereas those in the murine mammary gland were 2, 65 and 33%, respectively (25). A study determined the major isoform of PFK-1 in breast cancer cells using western blot analysis. PFK-P was identified to be the major isoform in breast cancer cells, including MCF-7, MDA-MB-231, BT-474 and SK-BR-3 cells; whereas PFK-L was the major isoform in MCF10A cells, which is a non-tumorigenic breast cell line. The study shows that PFK-P plays a crucial role in the glycolytic activities and proliferation of breast cancer cells (26). However, the results of the study by Zancan *et al*(14) are inconsistent with these findings. The mRNA level of PFK-1 isoforms were also detected in three cell lines, MCF10A, MCF-7 and MDA-MB-231. The results revealed that the glycolytic efficiency in breast cancer cells depended primarily on the preferential expression of PFK-L over the PFK-M and PFK-P isoforms (14). The differences may be associated with the various aspects of PFK-1 expression and the different experimental conditions that were used, which may also indicate the complexity of the post-transcriptional regulation of PFK-1.

As previously described, the subunit composition has been shown to promote kinetic and regulatory differences among the isoenzyme pools that affect the affinity for fructose-6-P and for certain effectors, including ATP, AMP or fructose-2,6-P_2_, and that were suggested to contribute to the characteristics of the glycolytic operation in particular tissues. PFK-M is inhibited by allosteric inhibitors, including citrate and ATP, whereas PFK-L and PFK-P are less sensitive to the inhibitory effect of these allosteric effectors and are more sensitive to fructose 2,6-bisphosphate, which is a potent activator (26). PFK-P may contribute to maintaining a high glycolytic status at an increased citrate level and sufficient ATP concentration. Although PFK-L accounts for a large proportion of the total PFK-1 levels in human breast paracancer tissues, the total PFK-1 content in paracancer tissues is markedly lower than THAT in cancer tissues. Therefore, the PFK-1 activity is higher in cancer tissues.

The present study detected the PFK-1 isoenzyme patterns in human breast cancer and paracancer tissues and identified that during the development of breast cancer, the enhancement of glycolytic activity depends primarily on the conversion of PFK-1, from PFK-L to PFK-P.

## Figures and Tables

**Table I:** Common features of the groups divided by TNM classification according to the NCCN's guildlines of 2012.

TNM classification	N	Mean age (years)	Hormonal activity	

			Menopausal	Menstruating
I	10	56	7	3
II	14	54	10	4
III	16	57	12	4

The common features were not significantly different. NCCN, National Comprehensive Cancer Network.

## Abbreviations

LA: lactic acid
LDH: lactate dehydrogenase
HK: hexokinase
PFK-1: phosphofructokinase-1
PK: pyruvate kinase
